# Malnutrition Diagnosis and Food Consumption in Subacute Post-Stroke Patients During Rehabilitation

**DOI:** 10.3390/nu16213589

**Published:** 2024-10-22

**Authors:** Mariacristina Siotto, Alessandro Guerrini, Carola Cocco, Marco Germanotta, Alessio Fasano, Valeria Cipollini, Laura Cortellini, Arianna Pavan, Sabina Insalaco, Erika Antonacci, Elisabetta Ruco, Rita Mosca, Adriana Graziosi, Piera Spatola, Maria Rosaria Malanga, Biagio Campana, Irene Giovanna Aprile

**Affiliations:** 1 IRCCS Fondazione Don Carlo Gnocchi ONLUS, 50143 Florence, Italy; aguerrini@dongnocchi.it (A.G.); ccocco@dongnocchi.it (C.C.); mgermanotta@dongnocchi.it (M.G.); afasano@dongnocchi.it (A.F.); vcipollini@dongnocchi.it (V.C.); lcortellini@dongnocchi.it (L.C.); apavan@dongnocchi.it (A.P.); sinsalaco@dongnocchi.it (S.I.); eantonacci@dongnocchi.it (E.A.); eruco@dongnocchi.it (E.R.); rmosca@dongnocchi.it (R.M.); agraziosi@dongnocchi.it (A.G.); pspatola@dongnocchi.it (P.S.); rmalanga@dongnocchi.it (M.R.M.); bcampana@dongnocchi.it (B.C.); iaprile@dongnocchi.it (I.G.A.); 2 Department of Science and Technology for Humans and the Environment, Università Campus Bio-Medico di Roma, 00128 Rome, Italy

**Keywords:** post-stroke, rehabilitation, malnutrition, food consumption, plate waste method, GLIM criteria

## Abstract

Background: Stroke survivors frequently encounter malnutrition, adversely impacting clinical outcomes. Nevertheless, malnutrition and food consumption in post-stroke patients have not been frequently assessed, and their correlation with rehabilitation outcomes remains inadequately explored. The objective of this observational study was to evaluate malnutrition at admission in these patients, assess food consumption during a six-week rehabilitation program, and analyze their correlation with rehabilitation outcomes. Methods: Subacute post-stroke patients were evaluated at admission (T0) and after a six-week rehabilitation treatment (T1). At T0, we assessed clinical and demographic characteristics, and we diagnosed malnutrition according to the Global Leadership Initiative on Malnutrition (GLIM) criteria. Weight, BMI, hematochemical parameters, and activities of daily living with the modified Barthel Index (mBI) were evaluated at both T0 and T1; recovery was registered as a change in the mBI (ΔmBI = mBIT1 − mBIT0). Patients’ food consumption was recorded through visual plate waste estimation of three meals a day, 5 days a week, for six weeks of hospitalization for rehabilitation. Results: A total of 109 patients completed the study (51 women, mean age 69 ± 11). According to the GLIM criteria, 105 of these patients were at risk of malnutrition, while 43 were malnourished, with 15 severely malnourished. Malnourished patients wasted more food, with respect to non-malnourished patients, as measured by visual plate waste of total meals (25 ± 17% vs. 15 ± 14%; *p* = 0.001) and reached a lower ΔmBI. A linear regression analysis found a significant correlation between the ΔmBI, the waste of a “second dish”, which contained mainly protein, and serum albumin at admission, even after controlling for age. Conclusions: Malnutrition assessed with the GLIM criteria at admission and food consumption are two important nutritional parameters to evaluate in post-stroke patients hospitalized for rehabilitation due to their association with recovery.

## 1. Introduction

Stroke is a neurological disease that is the second most common cause of mortality worldwide and it is the leading cause of disability in adulthood [1]. This condition, which mostly affects those aged 65 and above, not only incurs substantial expenses for patients, families, and healthcare systems but also presents challenges for medical professionals due to its expected rise among the elderly population.

Malnutrition, specifically undernutrition, is defined as lack of nutrient intake, which implicates an altered body composition and body cell mass, with consequent lower physical and mental function, as well as lower clinical outcomes after diseases [2]. The prevalence of malnutrition in hospitalized patients differs significantly across healthcare settings. Malnutrition has a high prevalence across all stages of stroke treatment, as shown in a recent meta-analysis of 78 studies [3]. Specifically, this meta-analysis reported that during the early subacute phase, which occurs between 7 days and 3 months after the injury [4], 37% of stroke patients experienced malnutrition, reaching up to 52% in those who had an impaired nutritional status [3]. In rehabilitation/subacute care, this rate reaches 29% [5]. Malnutrition has a detrimental effect on clinical outcomes and mortality, but also on overall healthcare costs in stroke survivors, as previously demonstrated [6,7]. Very recent studies [8,9,10] and a recent systematic review and meta-analysis [11] demonstrate that malnutrition is also associated with poor functional outcomes after stroke, even if these studies are exiguous. Furthermore, very recently, one study reported that the presence of malnutrition, diagnosed with the Global Leadership Initiative on Malnutrition (GLIM) criteria, is associated with a lower recovery of activities of daily living in stroke survivors [12].

During rehabilitation treatment, which can last several months, it is critical that the patient’s nutritional status does not worsen. After a correct diagnosis of malnutrition at admission in a rehabilitation center, it is important to appropriately track stroke patients’ nutritional status during the rehabilitation period by regularly monitoring their dietary intake. In fact, this procedure allows us to detect nutritional deficiencies, to evaluate the risk of a patient’s nutritional status, and to start or track the effectiveness of nutrition care plans.

Visual estimation methods are straightforward and convenient techniques for estimating food consumption, allowing us to record the food or liquid consumed by subjects in different community contexts, such as a school or hospital [13,14,15,16,17]. They can be used as an alternative to the weighted method, which is the most precise but is also very time-consuming [18]. Among visual estimation methods, the “plate waste method” is often employed; it requires raters to estimate, by directly viewing the plate, how much food subjects/patients have consumed to the nearest increment on a scale from 0 to 100%, typically with a 25% increment. Several scales have been used, including 7-, 6-, 5-, 4-, and even 3-point scales, and they have been validated against weighted waste, yielding reasonably accurate approximations [18]. This method allows registering of food consumption among a discrete number of individuals in complex settings, such as hospitals or in rehabilitation settings for long-term care [19]. However, very few studies have investigated food consumption in rehabilitation settings [20].

To our knowledge, there is insufficient information regarding malnutrition, food consumption, and their correlation with recovery in post-stroke patients admitted to rehabilitation settings. In 2022, our group published preliminary data examining nutritional status and food consumption in a cohort of post-stroke patients suffering from sarcopenia, showing a correlation of these parameters with recovery [21].

Therefore, this study aims to evaluate the nutritional status in post-stroke patients, particularly by evaluating malnutrition with the GLIM criteria and the patients’ food consumption during the rehabilitation period. We then investigate the relationship between malnutrition, food consumption, and the rehabilitation outcome, namely the recovery of functionality in activities of daily living (ADLs).

## 2. Materials and Methods

### 2.1. Study Design and Participants

We designed an observational longitudinal study to recruit subacute post-stroke patients who were admitted to two rehabilitation centers of Fondazione Don Carlo Gnocchi, namely “S. Maria della Provvidenza” in Roma (RM, Italy) and “Polo Specialistico Riabilitativo” in S. Angelo dei Lombardi (AV, Italy), during the period from September 2020 to April 2023. The protocol was registered on ClinicalTrials.gov (brief title: NUTRISTROKE; identifier number: NCT04923165).

The inclusion criteria were as follows: first hemorrhagic or ischemic stroke diagnosed using computed tomography (CT) or magnetic resonance imaging (MRI), age between 18 and 85 years, time since stroke onset of less than six months, and sufficient cognitive and language abilities to sign the informed consent and comprehend all of the instructions.

The following were the exclusion criteria: previous stroke, and behavioral or cognitive disorders and/or reduced compliance interfering with active rehabilitation treatment or with understanding and giving informed consent.

The study protocol was approved by the Ethical Committee of Don Carlo Gnocchi Foundation, Milan, Italy, on 14 October 2020 (FDG_6_14/10/20). All patients provided written informed consent after receiving a thorough description of the study’s objectives.

### 2.2. Rehabilitation Treatment

Patients underwent a six-week rehabilitation program that included standard physical therapy for 45 min, six days a week. Active, active assisted, and passive mobilizations were used in rehabilitation, as well as muscle strength recovery exercises, stretching exercises, sensorimotor stimulation, postural passages and transfers, proprioceptive exercises, motor coordination and balance training, and walking, sitting, and standing training. Patients also performed task-oriented exercises such as reaching and grasping movements (e.g., reaching and picking up a glass or other objects) and activities of daily living (e.g., transfers, dressing, brushing/combing hair, depending on subject’s ability) to improve neuroplasticity and promote upper limb motor recovery.

Each patient also received daily upper limb robotic therapy five times a week for 45 min. Four technological devices were used in the robotic treatment: (i) Amadeo (Tyromotion, Graz, Austria), a robot that allows for passive, active, and active assisted finger flexion and extension movements; (ii) Pablo (Tyromotion, Graz, Austria), a sensor-based system that allows for unsupported three-dimensional shoulder, elbow, and wrist joint movements, both unilateral and bilateral; (iii) Diego (Tyromotion, Graz, Austria), which is an arm–shoulder robotic system that allows for three-dimensional, both unimanual and bimanual, movements of the shoulder joint, with arm weight support; (iv) Motore (Humanware, Pisa, Italy), a robotic device that allows for passive, active, and active assisted planar shoulder and elbow movements. Patients completed physical and cognitive tasks while receiving visual and audio feedback from upper limb robotic devices [22,23,24].

### 2.3. Clinical Assessment

Demographic, anamnestic, and clinical data were recorded at admission (T0). The comorbidities and severity were described according to the Cumulative Illness Rating Scale-Comorbidity Index, CIRS-CI, and the Cumulative Illness Rating Scale-Severity Index, CIRS-SI [25]. The presence of dysphagia was recorded at T0 based on clinical evaluation.

### 2.4. Nutritional Status Assessment

#### 2.4.1. Anthropometric Measurements

Anthropometric measurements were assessed as follows: Height was recorded at T0 for each patient able to stand, reporting data in meters (m). The height of subjects unable to stand was measured with knee height, using the Chumlea equation [26].

Body weight was recorded both at T0 and T1. Patients able to stand were weighed on a calibrated scale (Seca 750, Seca, Hamburg, Germany); patients unable to stand were weighed on a chair weighing scale (Wunder DE5, Wunder Sa.Bi. srl; Milan, Italy). Weight data were reported in kilograms (kg), accurate to the nearest 0.1 kg. The body mass index (BMI, kg/m^2^) at T0 and T1 was then calculated.

#### 2.4.2. Diagnosis of Malnutrition According to GLIM Criteria

We screened patients at T0 for risk of malnutrition by administering the Mini Nutritional Assessment Short-form (MNA-SF^®^), which is a quick and easy-to-use questionnaire validated as a stand-alone screening tool based on the full Mini Nutritional Assessment (full MNA^®^) [27]. The MNA-SF^®^ includes six geriatric-specific assessment questions on nutritional and health conditions. Patients were categorized as having a “normal nutritional status” if their MNA-SF^®^ screening score fell between 12 and 14, having a “risk of malnutrition” if it fell between 8 and 12, and being “malnourished” if it fell below 7.

After the screening step, the diagnosis of malnutrition at admission was made when patients had at least one phenotypic plus an etiologic criterion, as reported in the consensus GLIM criteria [2]. The phenotypic criteria adopted were as follows: (i) weight loss >5% within the past 6 months or >10% beyond 6 months; (ii) a low BMI, <20 if <70 years or <22 if >70 years; (iii) reduced muscle mass assessed by Bioelectrical Impedance Analysis (BIA, Akern, Italy), registering the Appendicular Skeletal Muscle Index (ASMI, kg/m^2^). The thresholds for reduced muscle mass were the following: <7 kg/m^2^ for men and <5.5 kg/m^2^ for women. The etiologic criteria considered for our patients were the presence of inflammation due to acute disease, injury, or chronic disease [2]. Malnutrition severity was assessed using a low body mass index (BMI) as phenotypic criteria to distinguish patients with moderate malnutrition (BMI < 20 if <70 years or BMI < 22 if ≥70 years) or severe malnutrition (BMI < 18.5 if <70 years or BMI < 20 if ≥70 years).

#### 2.4.3. Hematochemical Analyses

The blood samples of patients were collected in the early morning (7:30–9:00 a.m.) after overnight fasting to standardize the assessment of those biochemical variables that are affected by the circadian cycle and food intake. Sera samples were separated by centrifugation (3000 rpm, 10 min, and 4 °C). They were then divided into 0.5 mL aliquots and rapidly stored at −80 °C. The subjects’ samples and reference samples were thawed just before the assay. All samples at T0 and at T1 were tested on an integrated analytical photometer (Free Carpe Diem, Diacron, GR, Italy) in duplicate, using a specific assay provided by Diacron (Diacron, GR, Italy).

Albumin was assessed by using a bromocresol colorimetric assay (normal levels: 2.5–5.5 g/dL for women and 3–6 g/dL for men; [28]). Glucose was assessed by using an oxidase/peroxidase system (normal levels: 70–105 mg/dL; [29]). Total cholesterol was measured by means of oxidation from cholesteroxidase to cholest-4-en-3-one (normal levels: <200 mg/dL [30]). HDL cholesterol was assessed by the conversion of the HDL portion into a quinone derivative (normal levels: >65 mg/dL for women, and >55 mg/dL for men; [31]). Triglycerides were tested by using a peroxidase-coupled method (normal levels: 40–165 mg/dL; [32]). We also calculated the cholesterol ratio, dividing total cholesterol by HDL cholesterol.

#### 2.4.4. Food Consumption by Plate Waste Method Registration

The meals consumed were formulated and prepared according to the Italian guidelines “National Recommended Energy and Nutrient Intake Levels for the Italian Population” (LARN, [33]). Lunch (from 12:00 to 13:30) and dinner (from 18:00 to 19:00) were served in our rehabilitation centers according to Italian customs and consisted of three distinct dishes known as the “first dish”, “second dish”, and “side dish”, as well as an additional dish containing fruit. The “first dish” consists mainly of carbohydrates, for example, cereals such as pasta, rice, or semolina, and is typically seasoned with legumes or vegetables, or includes both. The “second dish” consists mainly of a protein source such as meat, fish, eggs, or dairy products. The side dish is composed of vegetables. The fruit offered was an entire piece of seasonal fruit (e.g., an apple, a banana, or an orange) or as a mousse. Meals for patients with dysphagia consisted of the same food, but with modifications made to the consistency, or liquids.

The amount of food wasted by patients during the 6 weeks of study was evaluated through the visual estimation “plate waste” method, as described previously [21,34,35]. Nurses and speech therapists recorded patients’ wasting of meals (breakfast, lunch, and dinner) for 6 days a week, for 6 weeks (108 meals in total for each patient). A score from 0 to 4 was assigned on a 5-point scale (0 = not wasted; 1 = ¼, 2 = ½, 3 = ¾, or all wasted). The daily average plate waste score was then calculated for all 108 meals consumed for all patients. Moreover, we separately analyzed the “first dishes” and the “second dishes” of each day. We, therefore, used the terms “total meals”, which combined data from all of the food consumed during the day, “first dish”, which combined data from the daily quantity of carbohydrates served in the first dishes of lunch and dinner, and “second dish”, which represented the total daily amount of protein consumed in the second dishes of lunch and dinner.

#### 2.4.5. Activities of Daily Living Assessment as Rehabilitation Outcome Measure

We assessed, both at T0 and at T1, the patients’ performance in ADLs, using the modified Barthel Index (mBI). It consists of 10 items that evaluate a patient’s ability to execute different activities (feeding, personal hygiene, dressing, bathing, bladder control, bowel control, toilet transfers, stair climbing, and ambulation/wheelchair). Based on the level of support required by the patient, each item is given a numerical value, resulting in a cumulative score of 100 points. Lower ratings reflect reduced levels of mobility and increased self-care impairment, whereas higher values suggest enhanced functional autonomy [36]. Recovery was calculated as the change in the mBI by subtracting the initial value of the mBI from the value reached by patients after rehabilitation (ΔmBI = mBIT1 − mBIT0).

### 2.5. Statistical Analysis

The demographic and clinical characteristics of the patients, along with plate waste data, were presented as the mean and standard deviation for continuous variables, and as counts and percentages for categorical variables. The normality of the data was assessed using the Shapiro–Wilk test, with subsequent use of parametric or non-parametric tests depending on the distribution.

At baseline, comparisons between women and men regarding demographic, clinical, and nutritional status were conducted using the Mann–Whitney U test for non-parametric data or the chi-squared test for categorical variables. The same statistical tests were applied to compare dysphagic versus non-dysphagic patients and malnourished versus non-malnourished patients, according to the GLIM criteria.

To assess food consumption differences between malnourished and non-malnourished patients, as well as between women and men, plate waste was compared using the Mann–Whitney U test. The Kruskal–Wallis test was later used to explore differences in food waste between non-malnourished, moderately malnourished, and severely malnourished patients based on GLIM malnutrition severity.

Post-rehabilitation changes in weight, BMI, and hematochemical parameters were analyzed separately for malnourished and non-malnourished patients using the Wilcoxon signed-rank test. Recovery in ADLs (ΔmBI) was compared between malnourished and non-malnourished groups using the Mann–Whitney U test.

The relationship between recovery, as measured by the ΔmBI, and malnutrition, food consumption, and clinical data was assessed using Spearman’s rank-order correlation or the Mann–Whitney U test, depending on the variable. A multiple linear regression analysis was then performed to examine the impact of food consumption on the ΔmBI, adjusting for confounding variables identified in the bivariate analyses. Multicollinearity was evaluated using Variance Inflation Factors (VIFs), with highly collinear variables removed as needed.

For all statistical analyses, a *p*-value below 0.05 was considered significant. Statistical analysis was performed using SPSS (IBM SPSS Statistics for Windows, Version 29.0. Armonk, NY, USA: IBM Corp.).

## 3. Results

### 3.1. Participants and Baseline Characteristic

We screened 140 patients: 119 were enrolled and 109 completed the study (51 women, mean age 69 ± 11).

Table 1 presents the baseline characteristics in terms of demographic and clinical features, ADLs, hematochemical analysis, and nutritional assessment reported for the whole group and disaggregated by gender. Gender differences were found in weight and height, as expected. Apart from age and HDL cholesterol, no other significant differences were found between the two genders.

There were 49 dysphagic patients, of which only 6 followed a restricted diet, eating only fluid food. In this study group, patients were not characterized by severe clinical conditions that required enteral (e.g., nasogastric tube, gastrostomy) or parenteral nutrition. Analyzing in detail dysphagic patients at admission, we found that they were older than non-dysphagic patients (72 ± 9 vs. 67 ± 12; *p* = 0.025) and had a lower weight (65.5 ± 17.4 vs. 72.1 ± 16.4; *p* = 0.005) and lower BMI (23.9 ± 4.8 vs. 25.6 ± 4.7; *p* = 0.025); moreover, they showed a lower mBI at T0 (34.4 ± 22.0 vs. 47.4 ± 20.1; *p*< 0.001).

### 3.2. Diagnosis of Malnutrition at Admission

**Figure 1:**
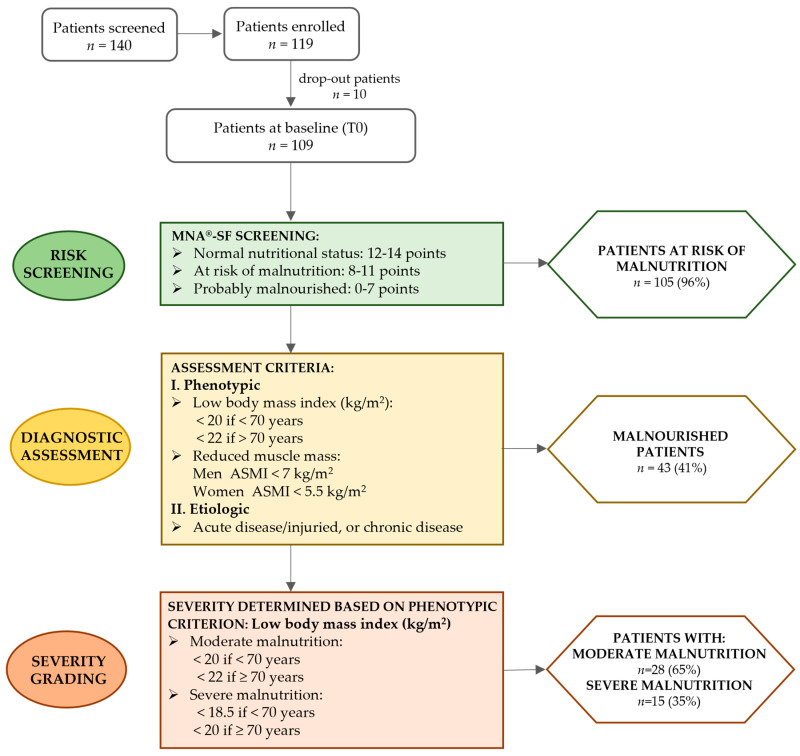
The diagnostic algorithm according to the GLIM criteria for case finding and the diagnosis of malnutrition at baseline in the enrolled patients. Risk screening (green boxes) was performed by using the Mini Nutritional Assessment Short-Form (MNA-SF^®^), thus finding patients at risk of malnutrition. The diagnosis of malnutrition (yellow boxes) was made by assessing the presence of at least one phenotypic criterion (I) and one etiologic criterion (II). The diagnostic criteria reported in the yellow rectangular box were those used for the sample group of the study. Finally, malnutrition severity (orange boxes) was assessed using the low body mass index as phenotypic criteria to distinguish patients with moderate or severe malnutrition.

We identified 105 subjects at risk of malnutrition, according to an MNA^®^-SF score below 11. Then, the diagnosis of malnutrition was made, finding that 43 individuals were malnourished due to the existence of at least one phenotypic and one etiologic criterion. The severity of malnutrition was evaluated and 28 were moderately malnourished, while only 15 subjects were found to be severely malnourished (Figure 1).

### 3.3. Differences Between Malnourished and Non-Malnourished Subjects at Admission

Dividing the sample group between malnourished and non-malnourished patients according to the GLIM criteria, we found no differences in terms of gender, age, time from stroke onset, CIRS comorbidities, CIRS severity, the number of dysphagic patients, or ADLs (Table 2). We found a difference in terms of the number of patients with hypertension, and an expected significant difference between two groups in terms of weight, BMI, and serum albumin concentration (Table 2).

### 3.4. Food Consumption During Hospitalization

**Figure 2:**
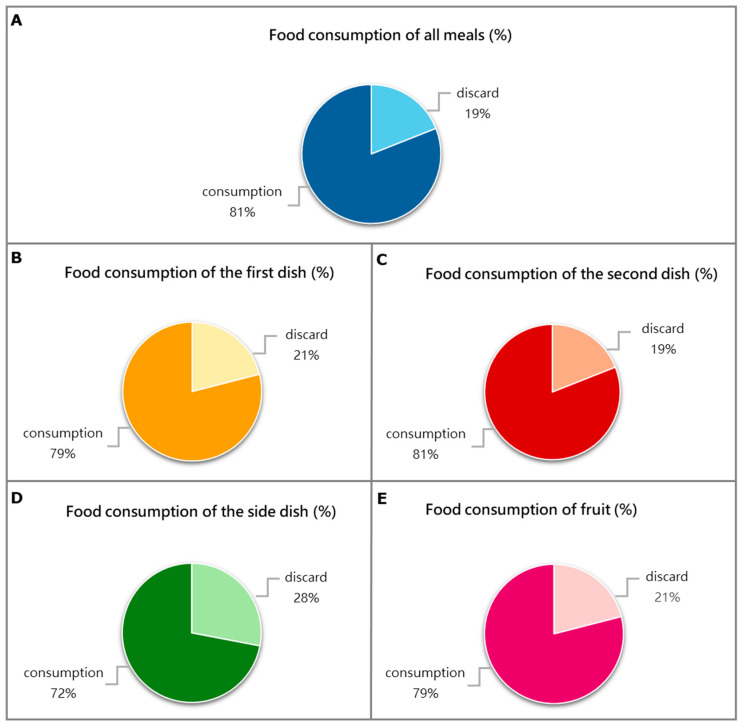
The pie charts display the percentages of daily average food consumption over the six weeks of treatment. Food consumption of meals served according to Italian customs is referred to as follows: (**A**) the totality of all meals; (**B**) the “first dish” (lunch + dinner) composed of carbohydrates, (e.g., cereals such as pasta, rice, or semolina), usually seasoned with legumes or vegetables, or both; (**C**) the “second dish” (lunch + dinner) made up of a protein source (meat, fish, eggs, or dairy products); (**D**) the “side dish” (lunch + dinner) which is usually vegetables; and (**E**) the fruit dish which consisted of a whole piece of seasonal fruit (e.g., an apple, a banana, or an orange) or as a mousse. The data are reported as the mean percentage (%). For dysphagic patients, meals were served with the same food modified in consistency or fluid.

At the end of the six weeks of observation, we analyzed the percentage of average daily food waste, considering the total meals served according to Italian habits, as reported in the Material and Methods Section (Figure 2). Patients on average wasted 19% of their total daily meals, 21% of the daily first dish, and 19% of the daily second dish; they also did not consume 28% of the daily side dish and 21% of the fruit.

**Figure 3:**
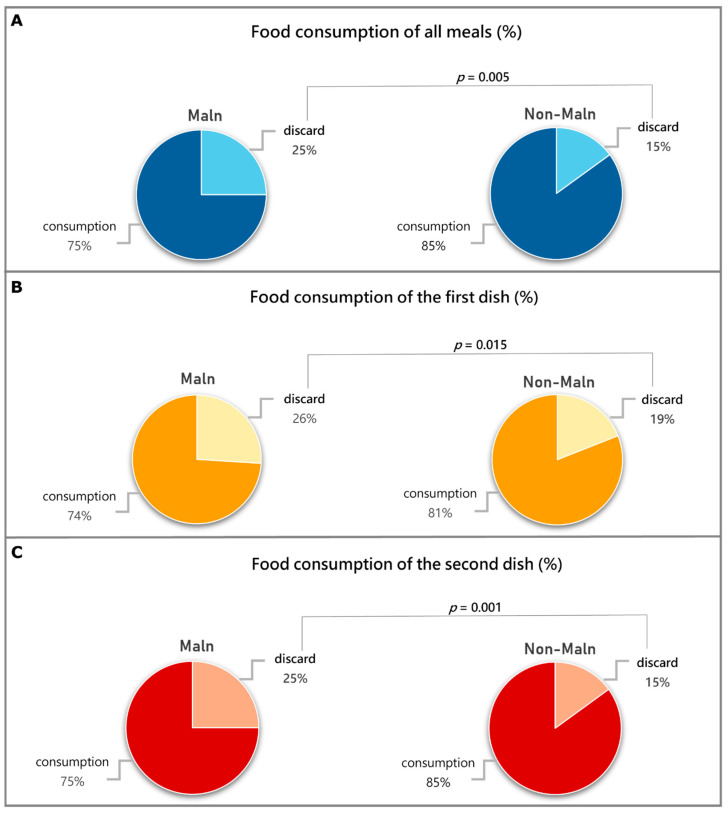
Differences in food consumption of meals served according to Italian customs (first dish, second dish, side dish, and fruit) between malnourished patients (Maln) and non-malnourished ones (non-Maln). (**A**) Food consumption of all daily meals expressed as the average percentage. (**B**) Food consumption of the “first dish” (lunch + dinner) expressed as the average percentage. (**C**) Food consumption of the “second dish” (lunch + dinner) as the average percentage. The *p*-value refers to the Mann–Whitney test.

Malnourished patients discarded more food with respect to non-malnourished patients in terms of the average of the “total meals”, “first dish”, and “second dish” (Figure 3). Women discarded more food with respect to men, specifically in terms of the “total meals” (21% vs. 17%; *p* = 0.038), “first dish” (25% vs. 18%; *p* = 0.011), and “second dish” (22% vs. 15%; *p* = 0.010). We did not observe differences for the side dish and fruit.

Moreover, dividing patients on the basis of malnutrition severity, the 15 severe malnourished patients discarded even more food, specifically more than 28% of the total meals (*p* = 0.005), 33% of the “first dish” (*p* = 0.004), and 29% (*p* = 0.001) of the “second dish”.

### 3.5. Malnourished Nutritional Status After Six-Week Rehabilitation Treatment

After the six-week rehabilitation program, malnourished post-stroke patients did not change weight and BMI (Table 3), while non-malnourished patients experienced a reduction in these parameters (Table 4). Among the hematochemical parameters, albumin levels significantly increased in malnourished patients.

### 3.6. Relationship of ADL After Rehabilitation with Malnutrition at Admission and Food Consumption

Malnourished patients achieved lower recovery in terms of the ΔmBI with respect to non-malnourished patients (13.3 ± 14.8 vs. 20.23 ± 18.3; *p* = 0.046).

There was a statistically significant, negative correlation between “total meals” and the ΔmBI (Spearman rho = −0.192, *p* = 0.048) and between the “second dish” and ΔmBI, (Spearman rho = −0.276, *p* < 0.004). The other dishes did not show any statistical correlation with recovery. Among clinical variables, only age (Spearman rho = −0.185, *p* = 0.05) and albumin (Spearman rho = 0.206, *p* = 0.037) showed a correlation with the ΔmBI.

The VIF analysis indicated that the waste of “total dishes” (VIF = 3.786) and the waste of the “second dish” (VIF = 3.859) had values above the suggested 2.5 threshold [35]. We included in the multiple linear regression analysis the waste of the “second dish” due to the higher correlation with the ΔmBI. As a result, only the waste of the “second dish” was analyzed. The multiple linear regression analysis showed a significant association between the ΔmBI, wasting the “second dish”, and albumin levels tested at admission even after adjusting for age (Table 5).

## 4. Discussion

The principal result of this observational study is that 41% of post-stroke patients admitted to our rehabilitation centers were malnourished, specifically undernourished, according to the GLIM criteria; 65% of these had moderate malnutrition, while 35% were severely malnourished. However, comparing malnourished and non-malnourished subjects, no differences were seen in terms of age, time from stroke event, the presence and severity of comorbidities, the presence of dysphagia, or ADLs. During the six-week rehabilitation program, malnourished individuals discarded more food than non-malnourished individuals, resulting in lower functional recovery in terms of ADLs as measured by the ΔmBI. Patients who are severely malnourished threw away even more food, up to 29% of “total meals” and the “second dish”, and up to 33% of the “first dish”.

To our knowledge, this is the first study to record food consumption in post-stroke patients across six weeks of rehabilitation. Moreover, it is worth noting that this study is one of the few that has identified malnutrition in post-stroke patients using the GLIM criteria.

An assessment of malnutrition using the GLIM criteria enabled the precise identification of persons who are really malnourished and the accurate characterization of their nutritional profile. The initial stage of diagnosing malnutrition includes assessing the risk of malnutrition using a validated screening test such as the MNA-SF^®^ [37], or other validated questionnaires such as MUST [38], NRS-2002 [39], and ESPEN 2015 [40]. These screening tools are particularly valuable for identifying patients with a nutritional status that classifies them as “at risk”. Nevertheless, in our dataset of stroke patients, the screening process unveiled that the vast majority (96%) of individuals were classified as “at risk” of malnutrition, as evidenced by a mean MNA-SF^®^ score below 11 points. Thus, MNA-SF^®^ screening accurately identified the risk of patients experiencing undernutrition during the subacute phase of stroke, which typically takes place from 7 days to 6 months after the initial event. Upon admission to a rehabilitation facility, patients have often experienced a previous hospitalization and may have hemiparesis, dysphagia, the presence of various comorbidities, and depression as a result of the stroke insult itself. All of these variables increase the probability of undernourishment, especially considering the older age of these persons. Nevertheless, the MNA-SF^®^ was unable to accurately identify the subjects who were suffering from ongoing malnutrition; therefore, it is essential to fully diagnose malnutrition using the GLIM criteria, which evaluate other significant phenotypic and etiologic parameters after the screening process [2]. This enables the precise identification of persons who are truly malnourished and the accurate characterization of their nutritional profile. The correct identification of the nutritional profile of patients allows physicians to monitor malnourished patients with greater precision and accuracy throughout their hospital stay for rehabilitation and recovery.

It should be noted that the correlation between malnutrition and the recovery outcome following a stroke has been poorly studied, save for a few recent analyses. Furthermore, the evaluation of nutritional status varies due to the absence of consensus on the most accurate diagnosis of malnutrition in post-stroke patients in rehabilitation settings [9,41].

Recent research showed that the GLIM criteria are s useful consensus tool to diagnose malnutrition in patients affected by stroke. It has been demonstrated that malnutrition assessed with the GLIM criteria can predict the recovery of ADLs in acute stroke patients [42]. Considering post-stroke individuals during rehabilitation, in our study, we found that malnourished subjects achieved lower recovery with respect to non-malnourished ones, and Kobayashi et al. [12] found an association between malnutrition diagnosed with the GLIM criteria and ADLs at discharge measured by FIM-motor score.

The experience gained from this study in diagnosing malnutrition with the GLIM criteria in post-stroke patients was instrumental in defining the nutritional status assessment of a multicenter study protocol in a large cohort of patients with stroke during rehabilitation treatment (trial registered at ClinicalTrials.gov with identifier NCT06547827).

Another important result of the study presented herein came from the analysis of food consumption. The totality of patients analyzed in our study discarded on average only 19% of all recorded meals. When we disaggregated subjects according to the GLIM criteria, we observed that malnourished individuals discarded a significantly greater amount of food. Moreover, individuals with severe malnutrition discarded up to 29% of “total meals”. Despite this, is important to notice that malnourished patients did not lose weight after six weeks of rehabilitation, and their hematochemical parameters did not worsen. Moreover, in malnourished subjects, albumin levels improved; considering that the half-life of albumin is about 3 weeks, this indicates that despite consuming less food, malnourished patients’ overall health did not worsen thanks to the clinical assistance provided during their hospitalization in our rehabilitation center. However, in this group of patients, the BMI remains almost unchanged during the weeks of observation.

We recorded food consumption using the “plate waste method”, which is a very practical and user-friendly “visual estimation method”. It has been shown to correlate with the weight method, which is the “gold standard” for tracking dietary intake but is also time-consuming and therefore impractical for routine clinical use [19]. On the other hand, “plate waste” monitoring appears to be a simple and efficient procedure to monitor patients’ food consumption in a hospital setting, where meals are provided directly from the canteen.

From the linear regression analysis, the recovery of post-stroke patients in terms of variations in ADLs (ΔmBI) appeared to correlate with the “second dish” and albumin levels at admission, even after correcting for age. This finding is particularly interesting, as it highlights the crucial significance of the nutritional “proteic” component in determining the likelihood of achieving positive functional recovery. The Italian second dish is principally made of protein, and albumin, which is the most abundant protein in human serum, has been used for decades as an indicator of malnutrition in patients in clinically stable conditions [43]. Prealbumin is currently the most favored biomarker for protein status assessment; however, the consumption of foods high in protein is directly correlated with albumin concentration. From these results, the importance of not only detecting albumin status at admission, but also detecting the consumption of protein-rich food during the rehabilitation period is elucidated due to their relationship with functional recovery. In line with our findings, other studies in the literature demonstrate that supplementation of amino acids exerted a positive effect on post-stroke patients’ recovery [44,45,46].

This study has certain limitations. Firstly, we did not record any bromatological data and were then unable to collect specific information on precise individual caloric intake. In order to further investigate this matter, future studies are needed.

The second limitation is that being an observational study, we could only record whether patients consumed food supplements. However, the formulations and doses of these supplements varied greatly, and this prevented us from drawing any meaningful conclusions about the effects of food supplementation. Future studies should be considered to determine whether specific supplementation (e.g., amino acids or other specific formulations) could be employed to sustain malnourished post-stroke patients during rehabilitation treatment. This could assist us in determining whether patients’ nutritional status can be raised during their rehabilitation.

A further limitation is the lack of detailed knowledge regarding the nutritional status of patients prior to the acute phase, including potential eating disorders that may have contributed to malnutrition or weight loss.

## 5. Conclusions

In conclusion, an accurate diagnosis of malnutrition at admission in post-stroke patients hospitalized for rehabilitation is of utmost importance [41], as undernutrition can have an impact on recovery. The GLIM criteria appeared useful in distinguishing malnourished from non-malnourished patients in such population. Moreover, as food consumption is associated with recovery, especially protein-rich meals, assessing food consumption using the visual plate waste method could be taken into account in a rehabilitation setting.

## Figures and Tables

**Table 1:** The baseline (T0) characteristics of the whole group (*n* = 109), of women (*n* = 51), and of men (*n* = 58). The data are reported as the mean ± standard deviation or number (%). *p*-values refer to the Mann–Whitney U test or the chi-squared test, as appropriate.

Baseline Characteristics	Whole Group (*n* = 109)	Women (*n* = 51)	Men (*n* = 58)	*p*-Value
Age (years)	69 ± 11	72 ± 9	67 ± 12	0.027 *
Index stroke type				
Ischemic	86 (79%)	42 (82%)	44 (76%)	0.407
Hemorrhagic	23 (21%)	9 (18%)	14 (24%)	
Affected side				
Right	58 (53%)	27 (53%)	31 (56%)	0.958
Left	51 (47%)	24 (47%)	27 (47%)	
Smoking habit (smokers and ex-smokers)	50 (46%)	19 (37%)	31 (53%)	0.103
Comorbidities				
Hypertension	93 (85%)	44 (86%)	49 (84%)	0.792
Type 2 Diabetes	28 (26%)	12 (24%)	16 (28%)	0.629
Dyslipidemia	45 (41%)	22 (43%)	23 (40%)	0.713
Heart disease	16 (17%)	4 (9%)	12 (23%)	0.059
Dysphagia	49 (45%)	24 (43%)	25 (47%)	0.679
Cumulative Illness Rating Scale (CIRS)				
CIRS severity	2.2 ± 0.4	2.2 ± 0.4	2.1 ± 0.4	0.066
CIRS comorbidity	5.1 ± 2.0	5.3 ± 1.9	4.9 ± 2.2	0.284
Days from stroke onset to enrollment	87 ± 57	97 ± 62	77 ± 52	0.079
Activities of daily living (ADLs) assessment				
modified Barthel Index (0–100)	42 ± 22	37 ± 19	46 ± 24	0.094
Hematochemical analyses				
Glucose (mg/dL)	111 ± 39	116 ± 45	105 ± 33	0.292
Cholesterol (mg/dL)	126 ± 36	133 ± 41	119 ± 31	0.207
High Density Lipoprotein (HDL) cholesterol (mg/dL)	53 ± 17	57 ± 15	49 ± 18	0.005 *
Ratio (cholesterol/HDL cholesterol)	2.5 ± 0.9	2.4 ± 0.8	2.6 ± 1.0	0.443
Triglycerides (mg/dL)	123 ± 58	120 ± 61	126 ± 56	0.372
Albumin (g/L)	3.8 ± 0.5	3.7 ± 0.5	3.9 ± 0.4	0.281
Nutritional status assessment				
Anthropometric value				
Weight (kg)	69.2 ± 17.1	62.2 ± 13.2	75.3 ± 17.9	<0.001 ***
Height (m)	1.66 ± 0.10	1.60 ± 0.67	1.71 ± 0.10	<0.001 ***
Body Mass Index (BMI; kg/m^2^)	24.9 ± 4.8	24.2 ± 5.1	25.5 ± 4.4	0.055

* *p*-value < 0.05; *** *p*-value < 0.001.

**Table 2:** Baseline (T0) differences in demographic, clinical, and nutritional status assessment of malnourished subjects (*n* = 43) and non-malnourished subjects (*n* = 66). Data are reported as mean ± standard deviation. *p*-values refer to Mann–Whitney U test or chi-squared *t*-test.

Baseline Characteristics	Malnourished (*n* = 43)	Non-Malnourished (*n* = 66)	*p*-Value
Gender, women	24 (56%)	27 (41%)	0.127
Age (years)	67 ± 12	72 ± 9	0.154
Anthropometric measurements			
Weight (kg)	57 ± 10	77 ±16	<0.001 ***
Height (m)	1.64 ± 10	1.67 ± 11	0.232
BMI (kg/m^2^)	21.0 ± 2.3	27.3 ± 4.3	<0.001 ***
Index stroke type			
Ischemic	35 (81%)	51 (77%)	0.606
Hemorrhagic	8 (19%)	15 (23%)	
Affected side			
Right	21 (49%)	37 (56%)	0.460
Left	22 (51%)	29 (44%)	
Smoking habit (smokers and ex-smokers)	17 (40%)	33 (52%)	0.231
Comorbidities			
Hypertension	32 (74%)	61 (92%)	0.009 **
Type 2 Diabetes	9 (21%)	19 (29%)	0.359
Dyslipidemia	15(35%)	30(45%)	0.273
Heart disease	4 (25%)	12 (75%)	0.200
Dysphagia	23 (53%)	26 (39%)	0.148
Cumulative Illness Rating Scale (CIRS)			
CIRS severity	2.2 ± 0.4	2.2 ± 0.4	0.270
CIRS comorbidity	5.3 ± 1.9	5.0 ± 2.1	0.259
Days from stroke onset to enrollment	93 ± 51	82 ± 61	0.075
Activities of daily living (ADL) assessment			
modified Barthel Index (0–100)	43 ± 20	41 ± 23	0.680
Hematochemical analyses			
Glucose (mg/dL)	113 ± 45	108 ± 35	0.717
Cholesterol (mg/dL)	126 ± 41	125 ± 33	0.779
HDL cholesterol (mg/dL)	57 ± 18	49 ± 15	0.037
Ratio (cholesterol/HDL cholesterol)	2.3 ± 0.9	2.7 ± 0.9	0.059
Triglycerides (mg/dL)	113 ± 42	130 ± 66	0.138
Albumin (g/L)	3.7 ± 0.5	3.9 ± 0.4	0.020 *

* *p*-value < 0.05; ** *p*-value < 0.01; *** *p*-value < 0.001.

**Table 3:** Changes in nutritional status and hematochemical parameters after rehabilitation in malnourished patients. Data are reported as mean ± standard deviation. *p*-values refer to statistically significant differences found by Wilcoxon signed-rank.

Malnourished (*n* = 43)	T0	T1	*p*-Value
Nutritional status			
Weight (kg)	57.0 ± 10.1	57.6 ± 11.1	0.404
Body mass Index (BMI; kg/m^2^)	21.0 ± 2.3	21.2 ± 2.7	0.400
Hematochemical parameters			
Glucose (mg/dL)	113 ± 45	105 ±43	0.216
Cholesterol (mg/dL)	126 ± 41	138 ± 52	0.218
High Density Lipoprotein (HDL) cholesterol (mg/dL)	57 ± 18	58 ± 16	0.346
Ratio (cholesterol/HDL cholesterol)	2.3 ± 0.9	2.5 ±1.0	0.638
Triglycerides (mg/dL)	113 ± 42	116 0 ±45	0.755
Albumin (g/dL)	3.7 ± 1.0	3.9 ± 0.5	0.041 *

* *p* < 0.05.

**Table 4:** Changes in nutritional status and hematochemical parameters after rehabilitation in non-malnourished patients. Data are reported as mean ± standard deviation. *p*-values refer to statistically significant differences found by Wilcoxon signed-rank.

Non-Malnourished (*n* = 66)	T0	T1	*p*-Value
Nutritional status			
Weight (kg)	77.1 ± 16.1	75.9 ± 15.5	0.002 **
Body mass Index (BMI; kg/m^2^)	27.4 ± 4.3	26.8 ± 4.1	0.001 **
Hematochemical parameters			
Glucose (mg/dL)	108 ± 35	103 ± 41	0.219
Cholesterol (mg/dL)	125 ± 33	124 ± 42	0.731
High Density Lipoprotein (HDL) cholesterol (mg/dL)	50 ± 15	51 ± 16	0.568
Ratio (cholesterol/HDL cholesterol)	2.7 ± 0.9	2.6 ± 0.8	0.361
Triglycerides (mg/dL)	130 ± 66	129 ± 64	0.977
Albumin (g/dL)	3.9 ± 0.4	4.0 ± 0.4	0.318

** *p* < 0.01.

**Table 5:** Multiple linear regression analysis for recovery (ΔmBI).

ΔBI	B	SE B	95% CI		*p*	R	R^2^	ΔR^2^
			LL	UL		0.373	0.139	0.113
Age	−0.179	0.153	−0.483	0.125	0.246			
Waste of “second dish”	−0.238	0.094	−0.4026	−0.051	0.013 *			
Serum albumin T0	7.341	3.632	0.133	14.548	0.046 *			

B: unstandardized regression coefficient; CI: confidence interval; LL: lower limit; UL: upper limit; SE B: standard error of coefficient; R: coefficient of determination R^2^: square of coefficient of determination; ΔR^2^: adjusted R^2^. * *p*-value < 0.05.

## Data Availability

The data supporting the findings of this study are available from the corresponding author upon reasonable request. Data are not publicly available due to privacy and ethical reasons.
